# 1,4-Bis[4-(dimethyl­silyl)phen­yl]benzene

**DOI:** 10.1107/S1600536810010883

**Published:** 2010-03-27

**Authors:** Lei Fang, Rui Wang, Li-Min Chen, Cai-Hong Xu, Shu-Hong Li

**Affiliations:** a Beijing National Laboratory for Molecular Sciences (BNLMS), Institute of Chemistry, Chinese Academy of Sciences, Beijing 100190, People’s Republic of China; b Graduate School of the Chinese Academy of Sciences, Beijing 100049, People’s Republic of China; c School of Chemical and Environmental Engineering, Beijing Technology and, Business University, Beijing 100037, People’s Republic of China

## Abstract

The complete molecule of the title compound, C_22_H_26_Si_2_, is generated by a crystallographic centre of symmetry. The central benzene ring makes a dihedral angle of 26.7 (4)° with the 4-(dimethyl­silyl)phenyl ring. There are weak C—H⋯π inter­actions in the crystal structure.

## Related literature

For applications of *p*-terphenyl derivatives as laser dyes, see: Craig *et al.* (1992 ▶), as light-emitting materials, see: Spiliopoulos *et al.* (2002 ▶) and as liquid crystalline materials, see: Yam *et al.* (1993 ▶). For a description of the Cambridge Structural Database, see: Allen (2002 ▶); Although *p*-terphenyls containing silyl groups have been reported (Feng *et al.*, 2007 ▶), their crystal structures have not yet been determined.
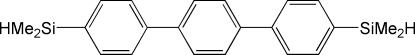


## Experimental

### 

#### Crystal data

C_22_H_26_Si_2_
*M* *_r_* = 346.61
Monoclinic, 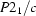

*a* = 15.143 (3) Å
*b* = 7.7263 (15) Å
*c* = 9.1285 (18) Å
β = 107.52 (3)°
*V* = 1018.5 (3) Å^3^
*Z* = 2
Mo *K*α radiation
μ = 0.18 mm^−1^
*T* = 173 K
0.20 × 0.18 × 0.08 mm

#### Data collection

Rigaku Saturn724+ CCD diffractometer
Absorption correction: multi-scan (*CrystalClear*; Rigaku, 2008 ▶) *T*_min_ = 0.966, *T*_max_ = 0.986
7661 measured reflections
2218 independent reflections
1937 reflections with *I* > 2σ(*I*)
*R*_int_ = 0.043

#### Refinement

*R*[*F* ^2^ > 2σ(*F* ^2^)] = 0.071
*wR*(*F* ^2^) = 0.133
*S* = 1.21
2218 reflections
115 parameters
H atoms treated by a mixture of independent and constrained refinement
Δρ_max_ = 0.31 e Å^−3^
Δρ_min_ = −0.22 e Å^−3^

### 

Data collection: *CrystalClear* (Rigaku, 2008 ▶); cell refinement: *CrystalClear*; data reduction: *CrystalClear*; program(s) used to solve structure: *SHELXS97* (Sheldrick, 2008 ▶); program(s) used to refine structure: *SHELXL97* (Sheldrick, 2008 ▶); molecular graphics: *ORTEPIII* (Burnett & Johnson, 1996 ▶); software used to prepare material for publication: *SHELXL97*.

## Supplementary Material

Crystal structure: contains datablocks global, I. DOI: 10.1107/S1600536810010883/fb2186sup1.cif

Structure factors: contains datablocks I. DOI: 10.1107/S1600536810010883/fb2186Isup2.hkl

Additional supplementary materials:  crystallographic information; 3D view; checkCIF report

## Figures and Tables

**Table 1:** Hydrogen-bond geometry (Å, °) *Cg*1 is the centroid of the C1–C6 ring.

*D*—H⋯*A*	*D*—H	H⋯*A*	*D*⋯*A*	*D*—H⋯*A*
C8—H8b⋯*Cg*1^i^	0.98	2.86	3.826 (3)	171
C10—H10a⋯*Cg*1^ii^	0.95	2.98	3.788 (3)	143

Symmetry codes: (i) 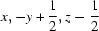; (ii) 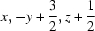.

**Fig. 1.**
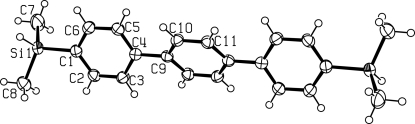
The title molecule, showing 50% probability displacement ellipsoids and the atom-numbering scheme. The atoms without labels are related to the labelled ones by -x, -y+1, -z+2.

**Fig. 2.**
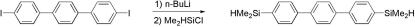
Reaction scheme for the synthesis of 1,4-bis(4-dimethylsilylphenyl)benzene

## supplementary crystallographic information

### Comment

*p*-Terphenyl derivatives have attracted considerable attention due to their extensive applications. Among others, they may be used as laser dyes (Craig *et al.*, 1992), light-emitting materials (Spiliopoulos *et al.*, 2002), liquid crystalline materials (Yam *et al.*, 1993). Although *p*-terphenyls containing silyl groups have been reported (Feng *et al.*, 2007), their crystal structures have not been given yet.

The title molecules are situated on the crystallographic centres of symmetry (Fig. 1). Two dimethylsilylphenyl-rings are linked to the central benzene ring in its 1,4 (*para*) positions. The rings are not coplanar; the dihedral angle between the dimethylsilylphenyl-ring and the central benzene ring equals to 26.7 (4)°. There are  C-H···π-electron ring interactions in the structure (Tab. 1).

The distance Si1-H1 (1.39 (3)Å) is in a fair accordance to the structures with the R-factor < 0.06 that have been retrieved from the Cambridge Crystallographic Database (version 5.31 from December 2009 with updates up to February 2010). (Allen, 2002). The average retrieved distance is 1.419 (14) Å for 21 observations. [The searched structures contained Si-fragment as it is in the title structure: 2 C atoms in sp^3^ state, 1 in sp^2^ state, 1 H. The structures with extensively deviated distances (ROLDIF, ROLDIF01, POZNEX, CODVOH, YOYBOD) have been suppressed.]

### Experimental

The reaction scheme is shown in Fig. 2. A solution of *n*-BuLi in hexane (1.6 M, 0.88 ml) was added dropwise to a solution of 1,4-bis(4-iodophenyl)benzene (192 mg, 0.40 mmol) in anhydrous tetrahydrofuran (THF) (80 ml) at -78 °C. After the solution having been stirred for 1 h, dimethylchlorosilane (152 mg, 1.60 mmol), also cooled to the same temperature, was added by syringe. The mixture was allowed to warm to room temperature and it was stirred overnight. After it had been quenched with saturated NaHCO_3_ solution, the mixture was extracted with Et_2_O. The organic layer was washed with brine, dried over anhydrous MgSO_4_, filtered, and concentrated under reduced pressure. The mixture was recrystallized from ethanol to give 92 mg of the title product in 67% yield. The crystals were colourless needles with the average length of about 3 mm.

### Refinement

All the hydrogens were discernible in the difference electron density maps. Nevertheless, all the hydrogens except the hydrogen attached to Si that was refined freely, were constrained by the riding-hydrogen formalism with U_iso_(H)=1.2U_eq_(C_aryl_) or U_iso_(H)=1.5U_eq_(C_methyl_). The C-H distances were constrained to 0.95 and 0.98 Å for the aryl and the methyl hydrogens, respectively.

### Crystal data

**Table:**

C_22_H_26_Si_2_	*F*(000) = 372
*M**_r_* = 346.61	*D*_x_ = 1.130 Mg m^−^^3^
Monoclinic, *P*2_1_/*c*	Mo *K*α radiation, λ = 0.71073 Å
Hall symbol: -P 2ybc	Cell parameters from 347 reflections
*a* = 15.143 (3) Å	θ = 2.2–27.5°
*b* = 7.7263 (15) Å	µ = 0.18 mm^−^^1^
*c* = 9.1285 (18) Å	*T* = 173 K
β = 107.52 (3)°	Plate, colorless
*V* = 1018.5 (3) Å^3^	0.20 × 0.18 × 0.08 mm
*Z* = 2	

### Data collection

**Table:**

Rigaku Saturn724+ CCD diffractometer	2218 independent reflections
Radiation source: fine-focus sealed tube	1937 reflections with *I* > 2σ(*I*)
graphite	*R*_int_ = 0.043
ω scans at fixed χ = 45°	θ_max_ = 27.0°, θ_min_ = 2.8°
Absorption correction: multi-scan (*CrystalClear*; Rigaku, 2008)	*h* = −19→19
*T*_min_ = 0.966, *T*_max_ = 0.986	*k* = −9→9
7661 measured reflections	*l* = −7→11

### Refinement

**Table:**

Refinement on *F*^2^	Primary atom site location: structure-invariant direct methods
Least-squares matrix: full	Secondary atom site location: difference Fourier map
*R*[*F*^2^ > 2σ(*F*^2^)] = 0.071	Hydrogen site location: difference Fourier map
*wR*(*F*^2^) = 0.133	H atoms treated by a mixture of independent and constrained refinement
*S* = 1.21	*w* = 1/[σ^2^(*F*_o_^2^) + (0.0276*P*)^2^ + 0.8805*P*] where *P* = (*F*_o_^2^ + 2*F*_c_^2^)/3
2218 reflections	(Δ/σ)_max_ < 0.001
115 parameters	Δρ_max_ = 0.31 e Å^−^^3^
0 restraints	Δρ_min_ = −0.21 e Å^−^^3^
46 constraints	

### Special details

**Table:**

Geometry. All esds (except the esd in the dihedral angle between two l.s. planes) are estimated using the full covariance matrix. The cell esds are taken into account individually in the estimation of esds in distances, angles and torsion angles; correlations between esds in cell parameters are only used when they are defined by crystal symmetry. An approximate (isotropic) treatment of cell esds is used for estimating esds involving l.s. planes.
Refinement. Refinement of *F*^2^ against ALL reflections. The weighted *R*-factor wR and goodness of fit *S* are based on *F*^2^, conventional *R*-factors *R* are based on *F*, with *F* set to zero for negative *F*^2^. The threshold expression of *F*^2^ > σ(*F*^2^) is used only for calculating *R*-factors(gt) etc. and is not relevant to the choice of reflections for refinement. *R*-factors based on *F*^2^ are statistically about twice as large as those based on *F*, and *R*- factors based on ALL data will be even larger.

### Fractional atomic coordinates and isotropic or equivalent isotropic displacement parameters (Å^2^)

**Table:**

	*x*	*y*	*z*	*U*_iso_*/*U*_eq_	
Si1	0.35065 (5)	0.48948 (10)	0.54899 (8)	0.0361 (2)	
C1	0.26293 (17)	0.4896 (3)	0.6572 (3)	0.0312 (5)	
C2	0.17225 (18)	0.4309 (3)	0.5939 (3)	0.0341 (6)	
H2A	0.1533	0.3895	0.4911	0.041*	
C3	0.10866 (17)	0.4313 (3)	0.6768 (3)	0.0338 (6)	
H3A	0.0474	0.3906	0.6299	0.041*	
C4	0.13394 (16)	0.4907 (3)	0.8282 (3)	0.0288 (5)	
C5	0.22485 (18)	0.5474 (4)	0.8935 (3)	0.0357 (6)	
H5A	0.2441	0.5869	0.9968	0.043*	
C6	0.28736 (18)	0.5469 (4)	0.8098 (3)	0.0365 (6)	
H6A	0.3487	0.5866	0.8572	0.044*	
C7	0.4368 (2)	0.3127 (5)	0.6203 (4)	0.0660 (11)	
H7A	0.4832	0.3164	0.5651	0.099*	
H7B	0.4051	0.2006	0.6028	0.099*	
H7C	0.4674	0.3283	0.7305	0.099*	
C8	0.2933 (2)	0.4691 (4)	0.3401 (3)	0.0468 (7)	
H8A	0.2475	0.5617	0.3060	0.070*	
H8B	0.2624	0.3565	0.3182	0.070*	
H8C	0.3398	0.4785	0.2854	0.070*	
C9	0.06529 (16)	0.4952 (3)	0.9166 (3)	0.0292 (5)	
C10	0.07421 (17)	0.6139 (3)	1.0364 (3)	0.0319 (6)	
H10A	0.1249	0.6924	1.0622	0.038*	
C11	0.01023 (17)	0.6186 (3)	1.1179 (3)	0.0322 (6)	
H11A	0.0178	0.7004	1.1985	0.039*	
H1	0.3948 (17)	0.649 (3)	0.578 (3)	0.036 (7)*	

### Atomic displacement parameters (Å^2^)

**Table:**

	*U*^11^	*U*^22^	*U*^33^	*U*^12^	*U*^13^	*U*^23^
Si1	0.0341 (4)	0.0455 (5)	0.0312 (4)	−0.0040 (3)	0.0137 (3)	0.0030 (3)
C1	0.0319 (13)	0.0320 (13)	0.0314 (12)	0.0010 (11)	0.0119 (10)	0.0015 (11)
C2	0.0384 (14)	0.0381 (14)	0.0266 (12)	−0.0009 (12)	0.0113 (11)	−0.0023 (11)
C3	0.0295 (13)	0.0366 (14)	0.0345 (14)	−0.0019 (11)	0.0085 (11)	−0.0027 (11)
C4	0.0300 (12)	0.0265 (12)	0.0298 (12)	0.0007 (10)	0.0086 (9)	−0.0002 (10)
C5	0.0359 (14)	0.0432 (16)	0.0281 (13)	−0.0026 (12)	0.0100 (10)	−0.0044 (11)
C6	0.0308 (13)	0.0426 (15)	0.0364 (14)	−0.0037 (12)	0.0106 (11)	−0.0023 (12)
C7	0.0479 (19)	0.094 (3)	0.065 (2)	0.0250 (19)	0.0313 (17)	0.026 (2)
C8	0.0528 (18)	0.0535 (19)	0.0370 (15)	−0.0004 (15)	0.0178 (13)	−0.0022 (13)
C9	0.0294 (12)	0.0298 (13)	0.0280 (11)	0.0030 (11)	0.0079 (9)	0.0045 (10)
C10	0.0295 (12)	0.0337 (13)	0.0319 (13)	−0.0033 (11)	0.0082 (10)	−0.0032 (10)
C11	0.0351 (14)	0.0331 (14)	0.0287 (12)	−0.0025 (11)	0.0101 (11)	−0.0037 (10)

### Geometric parameters (Å, °)

**Table:**

Si1—C8	1.848 (3)	C6—H6A	0.9500
Si1—C7	1.865 (3)	C7—H7A	0.9800
Si1—C1	1.879 (2)	C7—H7B	0.9800
Si1—H1	1.39 (3)	C7—H7C	0.9800
C1—C2	1.395 (3)	C8—H8A	0.9800
C1—C6	1.401 (3)	C8—H8B	0.9800
C2—C3	1.393 (3)	C8—H8C	0.9800
C2—H2A	0.9500	C9—C11^i^	1.401 (3)
C3—C4	1.396 (3)	C9—C10	1.402 (3)
C3—H3A	0.9500	C10—C11	1.388 (3)
C4—C5	1.396 (3)	C10—H10A	0.9500
C4—C9	1.496 (3)	C11—C9^i^	1.401 (3)
C5—C6	1.384 (3)	C11—H11A	0.9500
C5—H5A	0.9500		
			
C8—Si1—C7	111.07 (16)	C1—C6—H6A	119.0
C8—Si1—C1	110.83 (12)	Si1—C7—H7A	109.5
C7—Si1—C1	110.34 (13)	Si1—C7—H7B	109.5
C8—Si1—H1	108.8 (10)	H7A—C7—H7B	109.5
C7—Si1—H1	109.7 (10)	Si1—C7—H7C	109.5
C1—Si1—H1	106.0 (10)	H7A—C7—H7C	109.5
C2—C1—C6	116.6 (2)	H7B—C7—H7C	109.5
C2—C1—Si1	123.04 (18)	Si1—C8—H8A	109.5
C6—C1—Si1	120.39 (19)	Si1—C8—H8B	109.5
C3—C2—C1	122.0 (2)	H8A—C8—H8B	109.5
C3—C2—H2A	119.0	Si1—C8—H8C	109.5
C1—C2—H2A	119.0	H8A—C8—H8C	109.5
C2—C3—C4	120.7 (2)	H8B—C8—H8C	109.5
C2—C3—H3A	119.7	C11^i^—C9—C10	117.8 (2)
C4—C3—H3A	119.7	C11^i^—C9—C4	121.1 (2)
C5—C4—C3	117.8 (2)	C10—C9—C4	121.1 (2)
C5—C4—C9	121.3 (2)	C11—C10—C9	121.1 (2)
C3—C4—C9	120.9 (2)	C11—C10—H10A	119.5
C6—C5—C4	121.0 (2)	C9—C10—H10A	119.5
C6—C5—H5A	119.5	C10—C11—C9^i^	121.1 (2)
C4—C5—H5A	119.5	C10—C11—H11A	119.4
C5—C6—C1	122.0 (2)	C9^i^—C11—H11A	119.4
C5—C6—H6A	119.0		
			
C8—Si1—C1—C2	17.6 (3)	C4—C5—C6—C1	−0.2 (4)
C7—Si1—C1—C2	−105.9 (3)	C2—C1—C6—C5	−0.7 (4)
C8—Si1—C1—C6	−163.7 (2)	Si1—C1—C6—C5	−179.4 (2)
C7—Si1—C1—C6	72.8 (3)	C5—C4—C9—C11^i^	−154.1 (2)
C6—C1—C2—C3	0.8 (4)	C3—C4—C9—C11^i^	26.7 (4)
Si1—C1—C2—C3	179.6 (2)	C5—C4—C9—C10	26.2 (4)
C1—C2—C3—C4	−0.2 (4)	C3—C4—C9—C10	−153.1 (2)
C2—C3—C4—C5	−0.7 (4)	C11^i^—C9—C10—C11	−0.1 (4)
C2—C3—C4—C9	178.6 (2)	C4—C9—C10—C11	179.7 (2)
C3—C4—C5—C6	0.9 (4)	C9—C10—C11—C9^i^	0.1 (4)
C9—C4—C5—C6	−178.4 (2)		

Symmetry codes: (i) −*x*, −*y*+1, −*z*+2.

### Hydrogen-bond geometry (Å, °)

**Table:**

Cg1 is the centroid of the C1–C6 ring.

**Table:**

*D*—H···*A*	*D*—H	H···*A*	*D*···*A*	*D*—H···*A*
C8—H8b···Cg1^ii^	0.98	2.86	3.826 (3)	171
C10—H10a···Cg1^iii^	0.95	2.98	3.788 (3)	143

Symmetry codes: (ii) *x*, −*y*+1/2, *z*−1/2; (iii) *x*, −*y*+3/2, *z*+1/2.
